# Soft Tissue Extramedullary Plasmacytoma Leading to Cauda Equina Syndrome: Case Presentation and Clinical Considerations

**DOI:** 10.7759/cureus.14056

**Published:** 2021-03-23

**Authors:** Alyssa M Pace-Patterson, Tuba S Mirza, Ann I Payne-Johnson

**Affiliations:** 1 Research, Alabama College of Osteopathic Medicine, Pensacola, USA; 2 Family Medicine, Bariatric Medicine-Non Surgical Weight Loss Care, Ascension Sacred Heart, Pensacola, USA

**Keywords:** cauda equina, plasmacytoma, chronic low back pain (clbp), red flags

## Abstract

Cauda equina syndrome (CES) is a rare neurological emergency that requires prompt diagnosis and immediate surgical intervention for the best potential patient outcome. CES results from the compression of spinal roots along the lower spine usually at the level of L2 or below. It typically presents with severe low back pain, pain radiating to lower extremities, motor weakness, sensory loss, saddle anesthesia, bladder and bowel dysfunction. It is most commonly caused by a large central intervertebral disc herniation or central canal spinal stenosis but can also occur on occasion from abscesses, neoplasms, and inflammatory conditions. If the patient's symptoms are overlooked and surgical intervention is delayed there is a risk for long-term damage to neurological function. Here, we will present a case of a 46-year-old female with a long-standing history of back pain that presented to her primary care office with worsening back pain symptoms as well as a new presentation of urinary incontinence. A prompt MRI confirmed CES and the patient was advised to report to the nearest ED. At the hospital, neurosurgeons performed a laminectomy and found a mass along L3 that was compressing the cauda equina and associated nerve roots. The pathology of the mass revealed an extramedullary plasmacytoma (EMP) that was later determined to originate from the right psoas muscle. The case provides insight into the patient's presentation of CES and the key differentiating factors that led the medical care team to order the appropriate work up and prevent the long-term complications associated with an untreated CES.

## Introduction

Cauda equina syndrome (CES) is most commonly caused by a herniated disc in the lumbar region but on rare occasions it can occur due to a neoplasm. In this case the patient presented with epidural spinal cord compression as a complication of a soft tissue extramedullary plasmacytoma (EMP). A plasmacytoma is a very rare, solitary, monoclonal plasma cell neoplasm. A diagnosis of a solitary plasmacytoma is confirmed by histological findings of clonal plasma cells at a single site with no other characteristics of multiple myeloma [1-3]. It can present as a solitary bone plasmacytoma (SBP) or EMP. EMPs represent less than three percent of all plasma cell neoplasms while SBPs are approximately five percent of all plasma cell neoplasms [1-3]. EMP is a rare neoplasm of soft tissue without involvement of bone marrow or other characteristics of multiple myeloma [2]. Approximately 80% of EMPs are associated with the mucosal area of the upper aerodigestive passages in the head and neck region [2]. Most of the symptoms arise from mass effect and compression of nearby structures. Neoplasms are a common cause of back pain however, approximately one percent of patients presenting to a primary care setting with a complaint of low back pain are diagnosed with a malignancy [4]. Here, we present a 46-year-old female with an atypical presentation of extramedullary soft tissue plasmacytoma in the setting of CES. This case report describes a slow onset CES in the presence of a rare extramedullary soft tissue plasmacytoma.

## Case presentation

A 46-year-old female presented to the primary care outpatient clinic with excruciating back pain radiating down to the lower extremities bilaterally along the inner calves. The patient presented to the office four weeks prior for back pain and was diagnosed with sciatica. She was prescribed tizanidine and tramadol to help alleviate her symptoms. Her past medical history was notable for chronic low back pain due to osteoarthritis diagnosed in 2017. She originally attributed her worsening pain to recently starting a new job where she was responsible for lifting a significant amount of weight, however, she reported that her back pain continued to worsen and described the pain as different from the previous pain. Additionally, the patient reported new onset urinary incontinence, numbness, and tingling in her inner thighs and calves. She reported that tramadol and tizanidine relieved her symptoms in the beginning, however, stopped providing any relief. The pain was described as constant and unrelieved by position or specific motions. She described the pain as sharp and tingling in nature. She reported the pain severity as a 9/10 at rest and after walking or movement, a 10/10.

Past medical history was significant for type 2 diabetes diagnosed in January 2020, essential hypertension diagnosed in July 2017, chronic back pain and osteoarthritis of the lumbar spine diagnosed in 2017, and iron-deficiency anemia diagnosed in August 2017. The patient had a cholecystectomy in 2015 without complications. The patient reports compliance with her medications: ergocalciferol (vitamin D2) 1,250 mcg capsule po once weekly, esomeprazole magnesium 40 mg po, losartan 100 mg tablet po daily, gabapentin 300 mg po daily, and ozempic 0.5 mg subcutaneous pen injection once weekly. Family history is significant for several family members having malignancy as illustrated below in Table 1. 

 

**Table 1:** Summary of the patient’s family history.

Relative	Medical history
Father	Hypertensive disorder, arthritis, heart disease
Mother	Hypertensive disorder, arthritis, kidney disease, anemia
Brother	Autoimmune disease, neoplasm of stomach
Maternal grandfather	Neoplasm of brain
Maternal uncle	Neoplasm of the bladder
Paternal uncle	Malignant tumor of the lung

**Figure 1:**
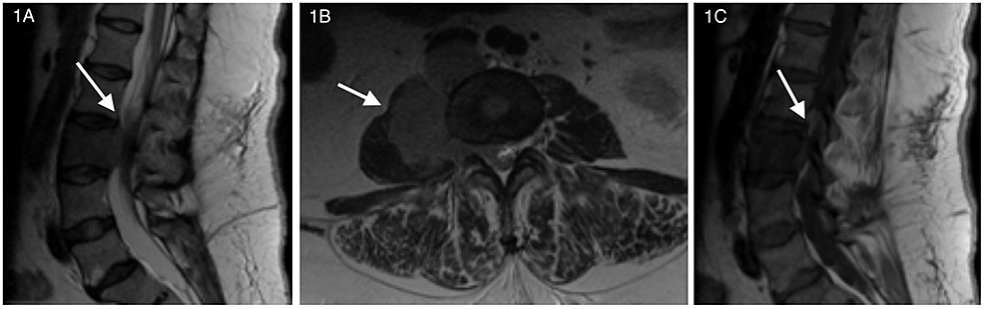
Noncontrast MRI of the lumbar spine. The image on the left (1A) is a sagittal T2 MRI of the lumbar spine, the middle image (1B) is an axial T2 MRI of the lumbar spine, and the image on the right (1C) is a sagittal T1 MRI of the lumbar spine. Interpretation: L3 vertebral body mass with prevertebral (7.5 cm x 4.2 cm x 6.1 cm) and epidural (2.1 cm x 1.2 cm x 4.5 cm) components. There is an evident retroperitoneal mass invading L3 vertebral body and extending into the epidural space with the epidural component of the mass resulting in severe compression of the thecal sac/cauda equina.

At arrival the patient was afebrile (98.4℉) and hypertensive (161/80), with a regular heart rate of 91, respiratory rate of 18, and oxygen saturation of 100%. Physical exam revealed a healthy-appearing, well-nourished patient in significant pain. Musculoskeletal exam revealed pain along the lumbosacral spine with all range of motion (ROM), pain with movement and reduced ROM by 80% (great difficulty with getting out of chair). Neurological exam was significant for sensory deficits along the inner thigh. Considering the new onset urinary symptoms and change in intensity and description of pain from prior encounters an urgent MRI was ordered, the imaging results are displayed in Figure 1. 

The patient was called and informed of her diagnoses and was instructed to immediately report to the nearest ED. Upon arrival to the ED, lab work and CT scan without contrast were ordered and neurosurgery was consulted (Table 2). CT scan confirmed a gently lobulated soft tissue mass arising from the right psoas muscle extending into right L3 neural foramen resulting in CES. The CT scan also determined that unlike the infiltrative changes seen in the recent MRI, there was no definitive destructive osteoblastic or osteolytic changes evident. 

**Table 2:** ED laboratory results at patient admission. WBC, white blood cell count; Hgb, hemoglobin; Hct, hematocrit

Laboratory type	Patient value	Reference value
WBC	10.6 K/uL	4.0-11.0 K/uL
Hgb	11.1 g/dL	Female 12.0-16.0 g/dL
Hct	32.5%	Female 36%-46%
Platelet	372 K/uL	150-400 K/uL
Sodium	137 mmol/L	136-146 mmol/L
Potassium	4.4 mmol/L	3.5-5.0 mmol/L
Creatinine	0.69 mg/dL	0.6-1.2 mg/dL
Calcium	8.9 mg/dL	8.4-10.2 mg/dL
Magnesium	2.1 mg/dL	1.6-2.6 mg/dL

Neurosurgery confirmed the diagnosis of CES and performed a laminectomy of L3. Additionally the tumor was removed and sent to pathology. Upon removal the tumor was described to be a very hemorrhagic, soft, and rubbery tissue that was highly attached to the dura. The biopsy was sent to pathology and the patient was discharged postoperative day one. Biopsy results confirmed the presence of a plasma cell neoplasm with the pathology report shown in Table 3 below. The differential diagnosis for the patient remained plasmacytoma versus multiple myeloma.

**Table 3:** Summary of the biopsy results.

Immunohistochemistry stain	Result
CD138	Positive
Cyclin D1	Negative
Congo red stain	Negative
Kappa/lambda light chains	1/500

Three days after discharge the patient followed up at her primary care office, where she reported a significant improvement in her symptoms. The patient’s back pain had improved drastically with only minimal residual urinary incontinence. Additional testing was ordered by medical oncology to assess for any additional neoplasms. After thorough evaluation no additional masses were found, kidney function remained adequate, and baseline anemia unchanged. Therefore, the final diagnosis of a plasmacytoma was confirmed. 

## Discussion

Multiple myeloma appears to be a systemic disease. In approximately five percent of cases it can present as an SBP or in approximately three percent of cases it can present as an EMP. These plasma cell neoplasms are differentiated based on the presence or absence of hypercalcemia, anemia, renal insufficiency, or bone marrow involvement. EMPs most commonly arise in the mucosal area of the upper aerodigestive passages. It is even more rare to find EMPs arising elsewhere. The diagnosis of a soft tissue EMP requires monoclonal plasma cells to infiltrate with no evidence of systemic disease [1-3]. An EMP usually presents with compressive symptoms of nearby structures. In this case the patient presented with epidural spinal cord compression as a complication of a soft tissue EMP. In approximately 20% of cases, epidural spinal cord compression is the initial manifestation of a malignancy [5]. It is not an uncommon presentation, however, it has been reported that only 11% of those cases are due to multiple myeloma [5]. A paraspinal mass extending into the epidural space was found in 10% of the cases with lymphoma found to be the most common cause [6-7]. One of the symptoms of epidural spinal cord compression is CES [6-7]. The case presented here showed a retroperitoneal mass in the psoas muscle invading the L3 vertebral body and extending into the epidural space. Pathology confirmed this mass to be a soft tissue EMP. The rarity of this case presentation is what prompted this case report. 

Cauda equina syndrome is a complex of low back pain, sciatica, saddle anesthesia, and lower extremity muscle weakness which progresses to bowel and bladder incontinence. Recognition of slow onset CES can be challenging as it can mimic chronic, recurrent low back pain with varying symptoms. In this case, the patient presented with early signs and symptoms suggestive of chronic mechanical low back pain with progression to neurological signs and eventually to the development of urinary incontinence completing the complex of CES. In this case report the patient had a three-year history of back pain prior to the onset of radicular signs. After three years of back pain, the patient presented with pain increasing in duration and intensity at each office visit. Additionally, the pain had progressively become less responsive to conservative treatment. 

The CES is most commonly caused by a herniated disc in the lumbar region and other causes include spinal lesions and tumors, spinal infections, lumbar spinal stenosis, trauma to lower back, birth abnormalities, spinal arteriovenous malformations, spinal hemorrhages, postoperative lumbar spine surgery complications, and spinal anesthesia [8]. CES can mimic other conditions and the symptoms can vary in intensity. Some of the conditions it can mimic includes peripheral nerve disorder, conus medullaris syndrome, spinal cord compression, and lumbosacral plexopathy [8]. As presented in this case, the mass effect of a tumor lesion was noted in the spinal canal and at existing nerve roots. 

Although one of the causes of CES is tumors, they are a very rare cause of low back pain. Approximately one percent of patients presenting to a primary care setting with a complaint of low back pain are diagnosed with malignancy [4]. The low prevalence of tumors causing low back pain can easily be the reason for not completing or initiating a malignancy workup in these patients. Most of the findings are incidental as in our case. Once “red flag” symptoms are identified there is an indication for MRI imaging. As presented through this case, the subsequent pathological work up of the mass revealed a plasmacytoma.

Back pain is the leading cause of disability worldwide and the second most common complaint encountered in primary care [9]. Patients and physicians should be aware of the “red flag” symptoms of severe low back pain: pain radiating to lower extremities, motor weakness, sensory loss, saddle anesthesia, bladder and bowel dysfunction, sexual dysfunction, and loss of deep tendon reflexes in the lower extremities [7]. Once these are identified, images such as MRI and myelogram can be helpful in diagnosing CES. If images suggest CES, urgent surgery is the treatment of choice [10-11].

## Conclusions

As one of the most common chief complaints presented at the primary care setting, low back pain can be easily overlooked. Although rare, one percent of back pain complaints in the primary care setting are later found to be due to a neoplasm and for this reason ought not be overlooked as a potential cause for low back pain. Frequent visits are scheduled with patients who present with worsening of their chronic symptoms, who fail to respond to previous conservative treatment, or who report onset of additional symptoms. Through these measures it will allow physicians to identify the “red flags” in a timely manner and prevent further complications. Additional studies need to be conducted to determine the most important red flag symptoms that would justify immediate imaging.

## References

[1] Dimopoulos MA, Kiamouris C, Moulopoulos LA (1999). Solitary plasmacytoma of bone and extramedullary plasmacytoma. Hematol Oncol Clin North Am.

[2] Fanning SR, Hussain MA, Perez-Zincer F (2006). Plasmacytoma, extramedullary. Emedicine.

[3] Vincent RS (2021). Diagnosis and management of solitary plasmacytoma of bone. UptoDate.

[4] Henschke N, Maher CG, Ostelo RW, de Vet HC, Macaskill P, Irwig L (2013). Red flags to screen for malignancy in patients with low-back pain. Cochr Datab Syst Rev.

[5] Schiff D, O'Neill BP, Suman VJ (1997). Spinal epidural metastasis as the initial manifestation of malignancy: clinical features and diagnostic approach. Neurology.

[6] Lada R, Kaminski HJ, Ruff R (1997). Metastatic spinal cord compression. Handbook of Clinical Neurology, Part III, 69.

[7] Posner JB (1995). Neurologic complications of cancer. Ann Neurol.

[8] Bagley CA, Gokaslan ZL (2004). Cauda equina syndrome caused by primary and metastatic neoplasms. Neurosurg Focus FOC.

[9] Traeger A, Buchbinder R, Harris I, Maher C (2017). Diagnosis and management of low-back pain in primary care. CMAJ.

[10] Korse NS, Pijpers JA, van Zwet E, Elzevier HW, Vleggeert-Lankamp CLA (2017). Cauda equina syndrome: presentation, outcome, and predictors with focus on micturition, defecation, and sexual dysfunction. Eur Spine J.

[11] Lavy C, James A, Wilson-MacDonald J, Fairbank J (2009). Cauda equina syndrome. BMJ.

